# Case of Ruptured Ectopic Pregnancy in the Uterosacral Ligament and Review of the Literature

**DOI:** 10.1155/2020/5897341

**Published:** 2020-08-11

**Authors:** Yasmin Abedin, Kanchi Chadha

**Affiliations:** ^1^Department of Obstetrics, Gynecology and Women's Health at Rutgers-New Jersey Medical School, Newark, New Jersey, USA; ^2^Department of Obstetrics and Gynecology at Hackensack University Medical Center, Hackensack, New Jersey, USA

## Abstract

Pregnancies that implant on the uterosacral ligament are rare. Here, we describe a case of ruptured ectopic pregnancy in the left uterosacral ligament in a patient with potential risk factors including possible endometriosis and recent hysteroscopic procedure. A 29-year-old female, para 0, presented to the emergency department with generalized abdominal pain. Pelvic examination was significant for fullness in the posterior cul-de-sac. Laboratory values were significant for beta-human chorionic gonadotropin (hCG) level of 6311 mIU/mL. Sonogram findings were significant for no intrauterine gestation, a 6.9 × 4.6 × 4.7 cm^3^ complex left adnexal mass, and moderate free fluid within the posterior cul-de-sac. The patient underwent laparoscopy, which revealed hemoperitoneum and unremarkable bilateral fallopian tubes and ovaries. An abnormal area was noted in the left uterosacral ligament. Tissue was bluntly removed and pathologically confirmed as chorionic villi within the left uterosacral ligament. After one week, her beta-hCG decreased to 784 mIU/mL. After two weeks, she was seen as an outpatient and was doing well without any symptoms. More information is required regarding these unique pregnancies to help understand the pathophysiology and determine the management.

## 1. Introduction

Pregnancies that implant on the uterosacral ligament are rare, and only a few other cases have been described in the literature. The incidence of these pregnancies ranges from 1 per 10,000 pregnancies to 1 per 30,000 pregnancies and approximately 1 per 100 ectopic pregnancies [1, 2]. These pregnancies present similar to tubal ectopic pregnancies and can be difficult to distinguish through ultrasonography; they can also be life threatening if not managed in a timely fashion. Here, we describe a case of ruptured ectopic pregnancy in the left uterosacral ligament in a patient with potential risk factors including possible endometriosis and recent hysteroscopic procedure. The pregnancy was managed with surgical intervention. Descriptions of such cases are needed in order to understand the pathophysiology of this disease and the best management guidelines. Moreover, we review other cases of ectopic pregnancies within the uterosacral ligament in order to gain insight into the risk factors and management of these pregnancies.

## 2. Case Presentation

This is a case of a 29-year-old female patient, para 0, who presented to the emergency department with worsening generalized abdominal pain. She had associated symptoms of fatigue and several episodes of diarrhea.

The patient's history was significant for infertility, for which she was undergoing evaluation at an outside institution. She had a hysterosalpingogram (HSG) about four months prior to presentation and three months prior to a hysteroscopy. Based on the HSG, there was suspicion for uterine polyps. One month prior to her presentation, she underwent a hysteroscopic procedure, which was uncomplicated. Two endometrial polyps were seen and removed, and bilateral tubal ostia were seen. She did not have a serum beta-human chorionic gonadotropin (hCG) test performed but urine beta-hCG was negative prior to the procedure. Her menstrual history was significant for irregular menses with intervals of 30-90 days. Her history otherwise was unremarkable.

**Figure 1:**
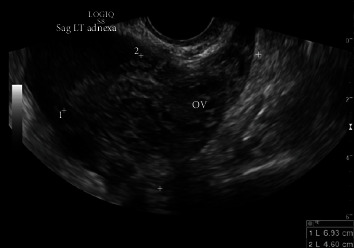
Transvaginal ultrasound image of left adnexa. OV depicts ovarian tissue. Complex left adnexal mass measuring 6.9 × 4.6 × 4.7 cm^3^.

On physical examination, the patient was pale and lethargic. She was tender on abdominal examination. Pelvic examination was significant for fullness in the right adnexa and posterior cul-de-sac. Laboratory values were significant for hemoglobin of 11.4 g/dL and beta-hCG level of 6311 mIU/mL. However, the patient was unaware of her pregnancy. Sonogram findings were significant for no intrauterine gestation, a 6.9 × 4.6 × 4.7 cm^3^ complex left adnexal mass, and moderate free fluid within the posterior cul-de-sac and right upper quadrant (Figure 1). The adnexal mass appeared to be heterogeneous, and the image was certainly ambiguous since no discrete gestational sac, yolk sac, or fetal pole were seen. However, in the setting of a positive pregnancy test and acute abdominal findings, the adnexal mass was concerning for a ruptured ectopic pregnancy with hemoperitoneum.

**Figure 2:**
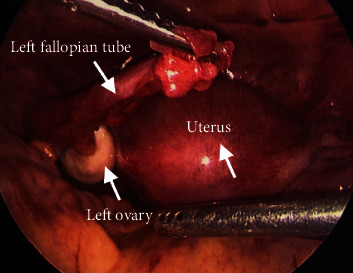
Laparoscopic image of unremarkable left fallopian tube and left ovary.

**Figure 3:**
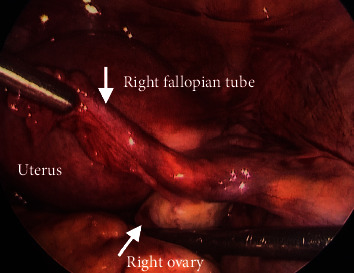
Laparoscopic image of unremarkable right fallopian tube and right ovary.

**Figure 4:**
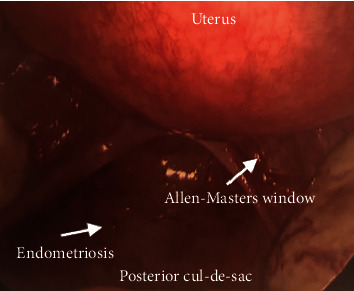
Laparoscopic image of posterior cul-de-sac, including endometriosis and Allen-Masters window.

**Figure 5:**
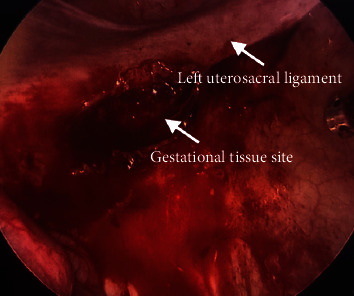
Laparoscopic image of left uterosacral ligament with the site of gestational tissue.

The patient underwent a diagnostic laparoscopy for evaluation and management of the suspected ectopic pregnancy. On laparoscopy, hemoperitoneum of 1000 mL was visualized and evacuated. The uterus appeared to be unremarkable. The fimbriated end of the left fallopian tube was minimally erythematous, but no evidence of rupture or injury was noted and the left ovary was unremarkable (Figure 2). The right fallopian tube and ovary were also within normal limits (Figure 3). The posterior cul-de-sac appeared to have adhesions and several areas of endometriosis, including the Allen-Masters window (Figure 4). Upon closer examination, the left uterosacral ligament had a 2 cm defect with abnormal tissue with bleeding (Figure 5). The tissue was suspicious for an ectopic pregnancy; hence, it was removed bluntly from the peritoneum and sent to pathology for assessment. Thereafter, this area of the left uterosacral ligament was copiously irrigated and suctioned. Persistent oozing of blood was observed, and SURGIFLO hemostatic matrix with thrombin was used to achieve excellent hemostasis.

At the end of the case, there was no identifiable pregnancy within the fallopian tubes or peritoneal cavity. The patient was extubated and had tolerated the procedure well. On postoperative day one, the patient did well and the repeat beta-hCG was 3807 mIU/mL.

Pathology results on postoperative day 2 revealed that the first specimen was a blood clot and that the second specimen was chorionic villi within the left uterosacral ligament. After one week, the patient's beta-hCG had decreased to 784 mIU/mL. After two weeks, the patient was seen as an outpatient and was doing well without complaints.

## 3. Discussion

Primary peritoneal pregnancy is a rare gynecologic morbidity. The incidence has been variable depending on the literature, ranging from 1 per 10,000 pregnancies to 1 per 30,000 pregnancies and approximately 1 per 100 ectopic pregnancies [1, 2]. The risks to the patient are similar as that of a ruptured tubal pregnancy; however, identification preoperatively is not trivial [2]. According to Studdiford, a primary peritoneal pregnancy needs to meet the following criteria: both fallopian tubes and ovaries appear to be normal without evidence of rupture or injury, absence of uteroperitoneal fistula, and the presence of a pregnancy related exclusively to the peritoneal surface and early enough to eliminate the possibility of secondary implantation [3]. We believe that our case met these criteria after laparoscopy was done and after pathology results confirmed chorionic villi within the peritoneum. However, the initial diagnosis was assumed to be a ruptured tubal ectopic pregnancy given the patient's presentation and unclear sonographic findings (Figure 1). The sonogram was ambiguous since no distinct gestational sac, yolk sac, or fetal pole were seen. However, an adnexal mass in the setting of a positive pregnancy test and acute abdominal findings was concerning for a ruptured ectopic pregnancy. Primary peritoneal pregnancies can have dire consequences, such as intraperitoneal hemorrhage with need for transfusion and even death. It is important for gynecologic surgeons to realize that the sonogram cannot always correctly diagnose a peritoneal pregnancy. There should also be a high index of suspicion for either ruptured tubal or abdominal pregnancy when a patient has a hemoperitoneum in the setting of a positive pregnancy test, and emergent laparoscopy is key to reduce morbidity. Furthermore, when an ectopic pregnancy is suspected and laparoscopy is initiated, it is imperative to perform a thorough pelvic and abdominal survey in order to locate the pregnancy implantation site.

There is a paucity of information regarding primary peritoneal pregnancies implanting in the uterosacral ligament, mostly because it is a rare entity. There have been few case reports on this topic (Table 1) [4–8].

Shin et al. described a 28-year-old G2P0010 patient with a ruptured ectopic pregnancy within the uterosacral ligament that was surgically removed via laparotomy. This patient did not have any apparent risk factors [4]. Lo and Lau described two cases; the first case was a 33-year-old female G1P0 with a history of pelvic endometriosis who had a ruptured ectopic pregnancy within the uterosacral ligament [5]. She was surgically managed with laparoscopy, which was converted to laparotomy [5]. The second case was a 32-year-old G4P2 with similar presentation, without any risk factors, managed with laparoscopic excision of abnormal tissue [5]. Gundabattula and Pochiraju described a 30-year-old G3P1011 with no significant risk factors with a ruptured ectopic pregnancy within the uterosacral ligament, which was managed with laparoscopy and one dose of parenteral methotrexate 50 mg/m^2^. There was a significant decline in her beta-hCG levels after one week (5699 mIU/mL to 81 mIU/mL) [6]. Cheung and Rosenthal discussed a 24-year-old female patient with similar presentation and no risk factors, managed with laparoscopy only [7]. Finally, Dasari and Devi reported a 22-year-old female G2P1 with a ruptured uterosacral ligament pregnancy, which was managed with laparotomy. An intrauterine device (IUD) was listed as a possible risk factor [8]. See Table 1.

The majority of these cases did not document any risk factor; however, pelvic endometriosis and IUD were listed as potential risk factors for having a primary peritoneal pregnancy within the uterosacral ligament. Our case did not have any of these documented risk factors prior to her procedure. She did not have any prior sonographic images depicting endometriosis and did not undergo laparoscopy previously. Based on our assessment during the laparoscopic procedure, it was evident that she did have endometriosis in the posterior cul-de-sac (Figure 4), which could have been a contributing risk factor to the peritoneal pregnancy within the left uterosacral ligament.

The pathophysiology of pregnancies that implant on the uterosacral ligament involves sperm that accumulates in the posterior cul-de-sac and ovum that also lies in the same region due to the physiologic flow of peritoneal fluid [9, 10]. Interestingly, hysteroscopy has not previously been described as a potential or definite risk factor for uterosacral ligament ectopic pregnancies, and it is remarkable that our case underwent this procedure just one month prior to presentation. It is possible that the hysteroscopic fluid could have assisted with patency of the fallopian tubes to allow for the sperm and ovum to meet in the peritoneum and implant in the uterosacral ligament, which can be considered a risk factor. On the contrary, one must also consider whether or not the hysteroscopic fluid could have facilitated the movement of an early tubal pregnancy into the peritoneum. However, the patient's urine beta-hCG was negative prior to her hysteroscopic procedure, so pregnancy was unlikely at that time. Also, as mentioned previously, we believe our case meets Studdiford's criteria and was a primary peritoneal pregnancy.

Management of pregnancies in the uterosacral ligament will vary depending on the patient's condition, gestational age, size of ectopic, and surgeon's experience. The use of surgery via laparoscopy [5–7] and laparotomy [4, 5, 8] has been described as above. Administration of methotrexate and/or intracardiac potassium chloride has also been described in the past for treatment of abdominal pregnancies [6, 10–13]. Not all of the prior reported cases of pregnancies in the uterosacral ligament trended beta-hCG levels [6]. This should be considered a crucial part of management, especially if the location of pregnancy or diagnosis of intra-abdominal pregnancy cannot be confirmed immediately. In our case, we successfully used laparoscopy and blunt removal of the gestational tissue from the uterosacral ligament and also followed beta-hCG values.

## 4. Conclusion

In summary, we reported a case of a ruptured ectopic pregnancy that implanted in the left uterosacral ligament. The patient was treated with surgical removal of gestational tissue, and she was followed postoperatively. Primary peritoneal pregnancies are rare, and pregnancies in the uterosacral ligament occur even more infrequently. Cases of such pregnancies should be reported in order for clinicians to understand the pathophysiology and determine the best management practices. Providers should have a high index of suspicion and be vigilant in performing laparoscopy/laparotomy in the setting of a positive pregnancy test and hemoperitoneum. It is imperative to perform a thorough pelvic and abdominal survey during surgery and obtain tissue for pathological analysis in order to determine a diagnosis.

## Figures and Tables

**Table 1:** Summary of prior cases with an ectopic pregnancy within the uterosacral ligament.

Authors	Shin et al. [4]	Lo and Lau [5]	Lo and Lau [5]	Gundabattula and Pochiraju [6]	Cheung and Rosenthal [7]	Dasari and Devi [8]	Present case
Age (years)	28	33	32	30	24	22	29
Gravidity and parity	G2P0010	G1P0	G4P2	G3P1011	Not reported	G2P1	G1P0
Serum beta-HcG (mIU/mL)	Not reported	Not reported	Not reported	5699 to 81 after one week	1654	Not reported	6311 to 3807 after procedure to 784 after 1 week
Risk factors	None	Endometriosis	None	None	None	Intrauterine device	Endometriosis and recent hysteroscopic procedure
Management	Surgical removal via laparotomy	Surgical removal via laparoscopy converted to laparotomy	Surgical removal via laparoscopy	Surgical removal via laparoscopy followed by parenteral methotrexate 50 mg/m^2^	Surgical removal via laparoscopy	Surgical removal via laparotomy	Surgical removal via laparoscopy

## References

[1] Atrash H. K., Friede A., Hogue C. J. (1987). Abdominal pregnancy in the United States: frequency and maternal mortality. *Obstetrics and Gynecology*.

[2] Hailu F. G., Yihunie G. T., Essa A. A., Tsega W. . (2017). Advanced abdominal pregnancy, with live fetus and severe preeclampsia, case report. *BMC Pregnancy and Childbirth*.

[3] Studdiford W. E. (1942). Primary peritoneal pregnancy. *American Journal of Obstetrics and Gynecology*.

[4] Shin J. S., Moon Y. J., Kim S. R., Kim K. T., Moon H., Hwang Y. Y. (2000). Primary peritoneal pregnancy implanted on the uterosacral ligament: a case report. *Journal of Korean Medical Science*.

[5] Lo K. W.-K., Lau T.-K. (1997). Ectopic pregnancy in uterosacral ligament. *The Journal of Obstetrics and Gynaecology Research*.

[6] Gundabattula S. R., Pochiraju M. (2014). Primary abdominal pregnancy in the uterosacral ligament with haemoperitoneum: a near miss. *Journal of Clinical and Diagnostic Research*.

[7] Cheung V. Y. T., Rosenthal D. M. (2005). Abdominal pregnancy. *Journal of Minimally Invasive Gynecology*.

[8] Dasari P., Devi S. (2000). Primary peritoneal pregnancy: a case report. *The Journal of Obstetrics and Gynaecology Research*.

[9] Cavanagh D. (1958). Primary peritoneal pregnancy. *American Journal of Obstetrics and Gynecology*.

[10] Agarwal N., Odejinmi F. (2014). Early abdominal ectopic pregnancy: challenges, update and review of current management. *The Obstetrician & Gynaecologist Gynaecologist*.

[11] Cobellis L., Stradella L., Messalli E. M. (2000). Contribution to the choice of therapy in abdominal pregnancy. *Panminerva Medica*.

[12] Anderson P. M., Opfer E. K., Busch J. M., Magann E. F. (2009). An early abdominal wall ectopic pregnancy successfully treated with ultrasound guided intralesional methotrexate: a case report. *Obstetrics and Gynecology International*.

[13] Deka D., Bahadur A., Singh A., Malhotra N. (2012). Successful management of heterotopic pregnancy after fetal reduction using potassium chloride and methotrexate. *Journal of Human Reproductive Sciences*.

